# Applying State-of-the-Art Deep-Learning Methods to Classify Urban Cities of the Developing World

**DOI:** 10.3390/s21227469

**Published:** 2021-11-10

**Authors:** A. K. M. Mahbubur Rahman, Moinul Zaber, Qianwei Cheng, Abu Bakar Siddik Nayem, Anis Sarker, Ovi Paul, Ryosuke Shibasaki

**Affiliations:** 1 Department of Computer Science and Engineering, The Independent University Bangladesh, Dhaka 1229, Bangladesh; akmmrahman@iub.edu.bd (A.K.M.M.R.); 1510190@iub.edu.bd (A.B.S.N.); 1521745@iub.edu.bd (A.S.); 1531144@iub.edu.bd (O.P.); 2 Data and Design Lab, Dhaka 1229, Bangladesh; zaber@unu.edu; 3 Department of Computer Science and Engineering, The University of Dhaka, Dhaka 1229, Bangladesh; 4 E-Government Operating Unit (UNU-EGOV), United Nations University, 4810-445 Guimarães, Portugal; 5 Center for Spatial Information Science, The University of Tokyo, Chiba 277-8568, Japan; shiba@csis.u-tokyo.ac.jp

**Keywords:** urban, categorization, building, planning, structures, sustainable

## Abstract

This paper shows the efficacy of a novel urban categorization framework based on deep learning, and a novel categorization method customized for cities in the global south. The proposed categorization method assesses urban space broadly on two dimensions—the states of urbanization and the architectural form of the units observed. This paper shows how the sixteen sub-categories can be used by state-of-the-art deep learning modules (fully convolutional network FCN-8, U-Net, and DeepLabv3+) to categorize formal and informal urban areas in seven urban cities in the developing world—Dhaka, Nairobi, Jakarta, Guangzhou, Mumbai, Cairo, and Lima. Firstly, an expert visually annotated and categorized 50 × 50 km Google Earth images of the cities. Each urban space was divided into four socioeconomic categories: (1) highly informal area; (2) moderately informal area; (3) moderately formal area, and (4) highly formal area. Then, three models mentioned above were used to categorize urban spaces. Image encompassing 70% of the urban space was used to train the models, and the remaining 30% was used for testing and validation of each city. The DeepLabv3+ model can segment the test part with an average accuracy of 90.0% for Dhaka, 91.5% for Nairobi, 94.75% for Jakarta, 82.0% for Guangzhou city, 94.25% for Mumbai, 91.75% for Cairo, and 96.75% for Lima. These results are the best for the DeepLabv3+ model among all. Thus, DeepLabv3+ shows an overall high accuracy level for most of the measuring parameters for all cities, making it highly scalable, readily usable to understand the cities’ current conditions, forecast land use growth, and other computational modeling tasks. Therefore, the proposed categorization method is also suited for real-time socioeconomic comparative analysis among cities, making it an essential tool for the policymakers to plan future sustainable urban spaces.

## 1. Introduction

Due to rapid globalization, many people have flown into urban spaces, accelerating the development of cities. According to UN DESA 2018 [1], more than 50% of the world’s population lives in urban areas. The growth rate is higher in developing countries in the global south. In order to keep the urban environment sustainable, policymakers need to plan based on extensive analysis of the urban environment. Automating categorization of formal and informal areas of living is vital for city planners and policymakers [2,3,4,5,6]. Categorization of urban building structures offers a number of benefits on health [7], education [8], and the environment [9]. The sustainable development goals of the United Nations emphasize the need for information about the physical environments of cities [10] as well as traffic conditions [11].

However, traditional tools, coupled with building categorization methods and models, are not oriented for developing countries where the surrounding environments are crucial for categorization. Moreover, these categorizations propose small-scale classifications, which give limited information [12], have poor scalability [13], are expensive due to the dependence on survey [14], and are also slow to compute in real-time [15]. With the advent of computational power, advanced image processing, and the availability of high-resolution satellite data, advanced machine learning, such as deep learning, is showing promise to solve the scalability problem.

Most recent studies on urban categorization are based on simple structural criteria, such as the types and conditions of individual buildings [16,17], building density, building height, and building scale as typical indicators (for example, [18,19]). However, the spatial structure and pattern of the urban environment are diverse and complex [13]. Specifically, automatic categorization of urban buildings in developing cities remains challenging due to various factors, such as building materials, surrounding environment, shabby structures, roofs made of cheap material, limited road access, high density, and varying building heights [20]. Unlike developed countries, these cities do not have dedicated residential areas, industrial areas, and pre-planned marketplaces. Hence, satellite images should ideally be segmented with certain structures, where the urban buildings are categorized meaningfully with their surrounding environment.

Manual categorization of a small area is neither economically viable nor scalable for large metropolitan cities, such as Dhaka, Mumbai, Lima, Jakarta, Guangzhou, Cairo, and Nairobi. The categorization scheme should facilitate automatic segmentation using state-of-the-art computational methods to ensure scalability at the lowest cost. Our extensive search failed to find research conducted on automatic urban categorization dedicated to developing cities. The traditional processes become costly and time-intensive to cover a wide range of cities. Moreover, due to the requirement of direct human intervention, we may not be very objective. To address these limitations, authors in [2] proposed a novel categorization method that shows promise in being readily usable by specialized deep learning models. This paper extends the work by applying the proposed categorization method at a large-scale over seven cities from developing countries. By extensive experiments, this paper shows that employing machine learning can help process larger data and index sets, ensure scalability, and reduce human-centric bias.

The categorization technique discussed in [2] helps to ascertain between informal and formal in urban environments. The categorization has advantages: we set the urbanization process and architectural form as two different axes instead of a single index. Then the physical environment of the city is divided into 16 categories. Through this classification method, all kinds of urban environmental forms can be summarized. Through manual operation, a large number of high-precision urban classification data involve studies where the scale is large: 50 × 50 km for each of the seven cities. Previous traditional studies were limited to small-scale classification, poor scalability, and were unable to provide a large amount of information.

The method of visual recognition based on [2] is used to detect and classify the physical environments of many cities and draw a map for the ground truth. The categorization technique includes buildings as well as their surrounding environments. Therefore, the texture and shape differences among various proposed categories are duly marked in the processed images. Therefore, this paper aims to extend the idea of the work [2] with a couple of contributions. We have applied three state of the art models (FCN-8 [21], U-Net [22], and DeepLabv3+ [12]) from different backbones. The textures and shapes are learned by corresponding models using the annotated training data set. Then each model was trained and tested over seven urban cities—Dhaka, Nairobi, Jakarta, Guangzhou, Mumbai, Cairo, and Lima. The training set encompasses 70% area of each of the cities, and the test set covers the rest 30%. In particular, in the experiments, we trained the deep models to semantically segment the satellite image of each city into four different urban areas, based on the buildings and their environments. Specifically, we segmented the cities automatically into (1) highly informal area; (2) moderately informal area; (3) moderately formal area; and (4) highly formal area. The experimental results suggest that each of the models can successfully capture the texture and shape differences and can segment the satellite images, according to the proposed categories, and DeepLabv3+ achieves the best performance. Hence, this paper provides the following contributions:

1. We show the efficacy of the categorization method proposed in [2], in seven cities from the developing world.
2. We show that three models from different backbones are successful in socioeconomic segmentation.
3. We present comparative results for three different models for all of the cities mentioned above.
4. We discuss the implication of the categorization method, along with deep learning techniques on policymaking in developing countries.

The proposed categorization method can be used as the basis of urban geography and demographic research, on a large scale. It can also be used as an indicator to compare among cities, after combining with data of topography, area, population density, income, education, etc., as the basis of research. The proposed categorization method for four socioeconomic areas helps DeepLabv3+ achieve the best performance among the three models. DeepLabv3+ obtains 90.0%, 91.5%, 94.75%, 82.0%, 94.25%, 91.75%, and 96.75% accuracy for Dhaka, Nairobi, Jakarta, Guangzhou, Mumbai, Cairo, and Lima, respectively.

The rest of the paper is organized as follows. In the next section, we discuss the theoretical background of urban building categorization and deep learning-based automatic methods. Then we describe our novel categorization model with sufficient examples. In the fourth and fifth sections, we discuss the deep learning method to semantically categorize the building categories automatically from satellite images. Then in the sixth section, we present our results and discuss the impact of the proposed methodology over policy outcome, followed by the conclusion.

## 2. Research Background

In this section, we discuss the theoretical definition of the urban environment. We provide detailed information on how urban environments are different in developing worlds. Then, we discuss different theoretical research studies and their applications, upon which we have built our model.

### 2.1. Urban Environment

Due to global economic, social, and political circumstances, the distinctive characteristics between urban and rural areas are blurring. This makes it very difficult to define urban and rural areas in a distinguishable way. A number of authors have defined an urban area on the basis of demography, the concentration of administrative bodies, infrastructure, and a diverse set of livelihood and income generation activities. Additionally, urban areas have been characterized by high population density with mixed-income scales when compared to other areas [23]. Some cities might be delineated by municipal boundaries. However, many urban areas are usually outlined by the presence of administrative structures, such as government offices, courts, and a relatively higher concentration of services, such as hospitals and financial institutions (e.g., banks). In an urban setting, the forms of sustenance and income generation activities are diverse and, unlike rural areas, not bound mainly to agricultural production and mining raw materials [24]. The “urban environment of the developing world: urbanization” generally refers to an increasing shift from agrarian to industrial services and distributive occupations. A significant rural-to-urban demographic shift taking place throughout the world is fueling this urban growth. One observation, as a result from rapid urban growth worldwide due to the influx of populations into urban areas, is that it has created immense pressure on the infrastructure and essential services of cities. This phenomenon is more pronounced in the developing world, and it has increased economic disparity in cities, which is manifested in the proliferation and expansion of slums in cities.

**Figure 1:**
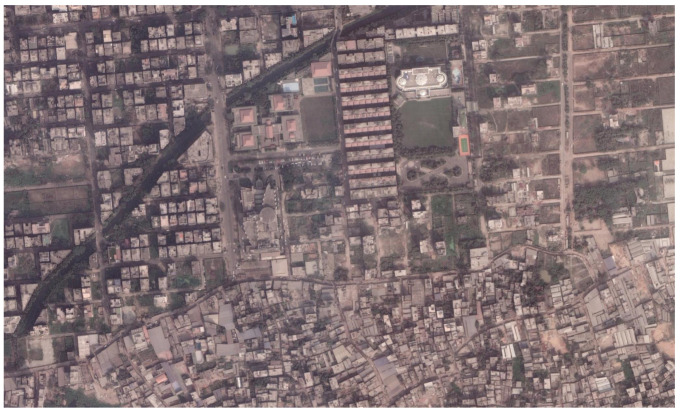
The morphological appearance of the urban environment in Dhaka.

For example, between 2003 and 2010, the city of Hyderabad in India experienced a 70% increase in slum areas [25]. Unfortunately, the situation is not unique to India or the global south. UN estimations show that the global slum population is around one billion at present and may rise to two billion by 2030 (UN-Habitat Report 2016 [26]). Among urban dwellers, many of them come to the city for jobs and start living in informal areas. Medium–high-wage job holders build their houses in better environments. However, they do not maintain the urban regulation and city corporation laws. Their areas might include shabby structures, as well as brick structures without any formal structures or surroundings. In this way, a moderately informal area is built. Figure 1 provides an example image of the urban environment in Dhaka.

The significant challenges for these developing cities include infrastructural services, basic amenities, environmental goods, environmental degradation, traffic jam and accidents, violence, and socioeconomic insecurity. Generally, these challenges are prevalent in highly informal and moderately informal areas. Understanding the socioeconomic conditions of different city–regions can be assisted by identifying different formal and informal settlements.

### 2.2. Traditional Machine Learning Techniques for Understanding Satellite Images

There are hundreds of earth observation satellites, providing data at different spectral, spatial, and temporal resolutions. ERS 1 and 2, Sentinel 1, 2, and 3 satellites launched by the European Space Agency (ESA), and Landsat 1–8 satellites, maintained now by the US National Oceanic and Atmospheric Administration (NOAA), have been providing satellite data (free of cost) for research. Commercial satellites, such as IKONOS, RapidEye, and WorldView 1–4 also provide much higher spatial (5 m) and temporal resolution satellite imagery, compared to that of Landsat and Sentinel (tens to hundreds of meters). As satellite images have visual channels (RGB), researchers apply spatial filters to analyze the textures and shapes of the satellite images to identify forests, water, buildings, farmlands, and meadows. In [27], the authors proposed a novel feature-extraction block extract spatial context of each pixel according to a complete hierarchical multilevel representation of the scene. Then they classified the features with a support vector machine(SVM). Cheng et al. [28] discussed traditional features, such as the histogram of ordered gradients (HOG), scale-invariant feature transform (SIFT), color histograms (CH), and local binary patterns (LBP), etc. Researchers use these features to segment the satellite images for different land covers by exploiting an SVM. Later, Sateesh et al. [29] developed a discrete wavelet-based image segmentation technique. The paper [30] developed a variational region-level criterion for supervised and unsupervised texture-based image segmentation. However, due to the high volume, and heterogeneity of satellite data, deep learning methods have been preferred over traditional manual or statistical methods to explore these datasets [31,32]. Deep learning methods show excellent performance in classification, image segmentation, as well as semantic segmentation.

### 2.3. Semantic Segmentation for Urban Environment Development

Technically, semantic segmentation involves classifying each pixel in an image from a predefined set of classes. It involves partitioning images (or video frames) into multiple segments or objects. Segmentation plays a central role in many applications, including medical image analysis (e.g., tumor boundary extraction and measurement of tissue volumes), autonomous vehicles (e.g., navigable surface and pedestrian detection), video surveillance, and augmented reality, to count a few.

**Figure 2:**
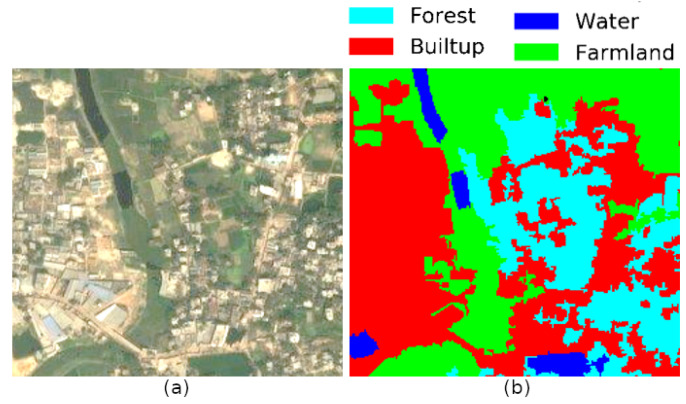
(**a**) Satellite image; (**b**) Land use land cover segmented image.

Earth observation satellites capture a whole range of data, from radio to visible, infrared, multispectral, and hyperspectral imagery. Since texture and shapes can be captured by deep-learning-based semantic segmentation, remote sensing researchers try to segment-specific areas based on different categorization tasks using one or more types of these data. The categorization tasks might range from land use land class (Figure 2) to slum detection, from tracking urban expansion to tracking forest degradation.

More recently, machine learning-based automated analysis of satellite imagery has been used to understand human socioeconomic conditions at a local (city, village, or country level) and global level. Both deep learning methods (e.g., convolutional neural networks (CNNs)) and shallow learning methods (e.g., support vector machines and random forests) are extensively used in predicting socioeconomic indicators, using daytime optical satellite imagery, radar data, and nighttime light (NTL) emissions captured by satellite sensors. However, to train such models, one needs both large quantities of satellite images, as well as non-satellite data from different sources, such as census, household and agricultural surveys, administrative data, and other environmental and meteorological data [33]. Machine learning methods have been used to estimate crop yields [34], land cover and land cover changes [32], slum detection [35], urban growth predicting and mapping poverty [36,37,38,39] population estimation [7], estimation of GDP [40], etc. Authors in [33] provide a comprehensive review of the potential use of earth observation data (both satellite and aerial imagery) to estimate socioeconomic indicators set by the UN’s sustainable development goals and the ’proxy’ indicators. Oshri et al. [41] used Afrobarometer [42] survey data along with satellite images from Sentinel 1 and Landsat 8 to assess infrastructure quality in Africa. They propose multi-channel inputs that utilize all available channels from respective satellites instead of only the RGB bands. Pre-trained ImageNet [43] weights have been used for the RGB channel, and the novel channels are initialized with Xavier Initialization [44]. A ResNet [45] based architecture was used with some minor modifications in their work, which achieves better AUROC results than nightlight-based works [38], OSM, and other baseline methods. Urban–rural split and country hold-out methods have been used to observe the model’s generalizing capabilities. The urban atlas [46] dataset, containing 20 land use classes across 300 European cities, has been used as ground truth, and is freely available, Google Maps image at zoom level 13, which translates to around ***1.20 m/px*** spatial resolution, as input images to identify patterns in urban environments by Albert et al. [47]. They contrasted two state-of-the-art networks, VGG-16 [48], and ResNet [45], and observed that ResNet-50 yields a slightly better result. Pre-training on ImageNet and DeepSat [49] in different settings was conducted to initialize the weights, then further fine-tuning was conducted on the prepared dataset. Urban environments across cities have been observed, since different classes can share similar features, and the same class can have different features across different sociocultural and economic zones. While predicting poverty from satellite images as a socioeconomic indicator of city areas, images from the DigitalGlobe [46] platform and Google Maps have been used as input imagery, where the spatial resolution used was 50 cm and 2.5 m, respectively [50].

## 3. Urban Environment Categorization

In this section, first, we briefly discuss the traditional urban environment categorization methods and their limitations in applications for developing countries. Then, we provide a summary of the novel categorization approach proposed in [2], which is suitable for deep learning applications.

### 3.1. Traditional Urban Environment Categorization

In the urban environment, different areas have distinct categories [2]. They are composed of different physical appearances, such as green spaces, artificially constructed, and naturally formed roads; regular, random, complex, dense buildings; tin-shed roofs, dilapidated structures; and high rise buildings with defined shapes. The study of urban morphology classification can provide an essential reference for the evolution and boundary division of historical regional morphology and provide in-depth understanding and training for practical application fields closely related to urban development, planning, architecture, urban management, and public policy. Barke et al. [51] pointed out that understanding urban morphology and its complexity is vital for urban development. Kärrholm et al. [52], and many other authors, point out the importance of understanding urban form as a critical element of urban sustainable development urbanization [3,4,53,54,55,56,57]. In the research methods of urban morphology taxonomy, Taubenbóck et al. [14] attempted to categorize according to historical/geographical methods and the physical density of buildings. Dovey et al. [15] considered the surrounding environment as well to study the urban morphology.

The body of studies devoted to analyzing cities in the developing world from the spatial scale is quite large. Huang et al. [12] used satellite images and cluster analysis to compare urban morphology. Combining high-resolution satellite data and auxiliary data sources [13] generates three-dimensional building models to obtain consistent and comparable urban data sets. However, the above-mentioned categorization methods were not custom-built to be used for modern machine learning techniques.

Several studies use deep learning better to understand urban areas from satellite images [41,42,58]. Oshri et al. [41] proposed to evaluate the environmental quality of Africa using [42] survey data and satellite images. They also use OpenStreetMap to identify the existence of different infrastructures. Neal et al. [58] used convolution neural networks (CNNs) to analyze the nighttime brightness of satellite images to estimate the economic activities in different regions. Albert et al. [47] proposed an in-depth learning method to identify patterns in an urban environment. Piaggesi et al. [50] used baseline VGG-16 (CNN model with 16 layers proposed by Visual Geometry Group) and ResNet-50 (Residual Network with 50 layers, proposed by Microsoft), pre-trained with ImageNet [43], and adjusted the luminous intensity label to predict the income of each family.

The complexity of the urban environment and the building structure is often closely related to its surrounding building environment, formation process, and geographical conditions. In urban research, a small area has been classified artificially. H. kamalipour et al. [59] used Google Earth data to divide the informal and formal areas of the urban environment. The method is practical and near accurate for small-scale projects. However, these classifications may not be enough to represent the inherent characteristics of cities in developing countries, nor can they better observe the changing trend between them. In addition, although these visual recognition methods are based on remote sensing images and have high recognition accuracy (in a small range), in many cases, they require a significant amount of time and necessary expertise.

### 3.2. Summary of the Urban Environment Categorization Method

Here, we provide the summary of the proposed categorization technique discussed in [2]. The categorization is based broadly on two dimensions—the state of urbanization and the architectural form of urban environment. Cheng et al. [2] categorizes an urban city into four semantic classes: (1) highly informal area; (2) moderately informal area; (3) moderately formal area; and (4) highly formal area using satellite images of the developing world.

**Figure 3:**
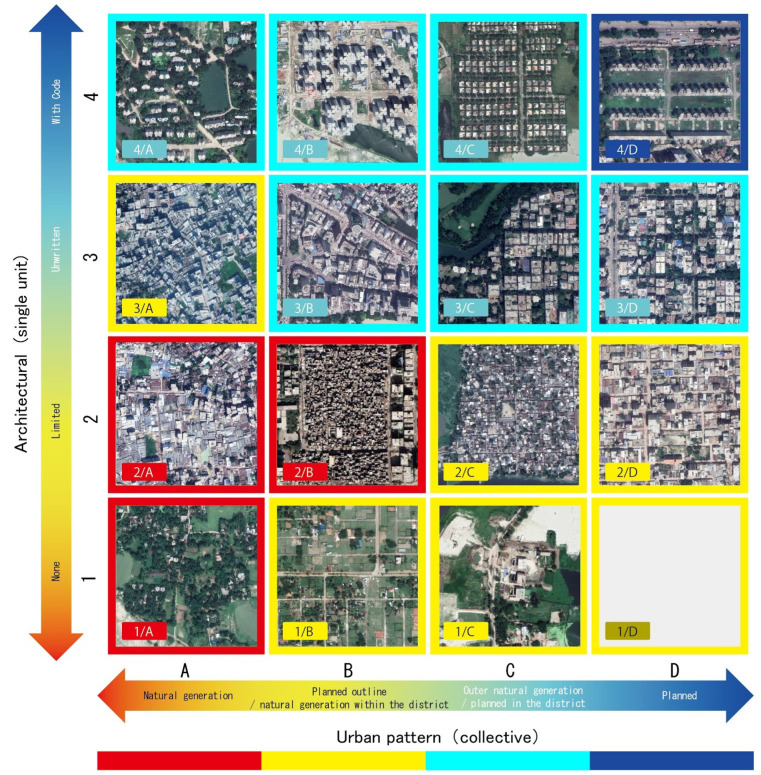
Urban Environment Topology for Cairo, this figure is adapted from [2].

It is generally believed that urban structures and buildings are the basic types of urban morphology. Researchers in [60], point out that the structure and form of traditional cities develop slowly, and there is no formal planning and design. Marshall and others also believe that the physical characteristics of streets, plots, and buildings are different. According to the above concepts, this study divides the urban natural environment into two indicators: vertical axis and horizontal axis Figure 3. The access network is reflected in urban planning, and architectural diversity is reflected in architecture. Authors in [2] divide the buildings into four categories: (1) category “none”; (2) category “limited”; (3) category “unwritten”; (4) category “coded”.

The indicator of the surrounding environment is also divided into four categories according to the regularity (natural generation or planned) of the access network inside and outside the building. The authors define the roads on the inside and outside as natural as “natural generation” marked as (A). Then, according to the regularity of the access network, the type of road network is defined as “planned outside + natural generation inside”, marked with letter (B). The type of road network with natural generation on the outside and the planned road network on the inside is defined as “natural generation outside + planned inside”, which is marked with the letter (C). Finally, we define the street network, which is planned both inside and outside as “planned” and mark it with a letter (D). Then, the four indicators of “building diversity” (marked with numbers 1, 2, 3, 4 from the bottom of the vertical axis) and the four indicators of “access network” (marked by the combination of letters A, B, C, and D on the left side of the horizontal axis). The obtained matrix is used as a standard criterion for categorizing every urban area with a scale of 400 m × 400 m as shown in Figure 3, where the combination of the two indicators forms 16 types. For Cairo, the 1/D type is not found (see Figure 3). That means public and tiny buildings are not present at the side planned street mode.

Now, by using the sixteen categories, the authors propose to segment metropolitan cities into four socioeconomic regions that semantically represent the socioeconomic conditions of those regions. The objective is to leverage the socioeconomic segmentation task to deep learning methods for large-scale applications. Please refer to the Table 1 for the complete list that defines the four socioeconomic areas, in terms of the sixteen basic categories.

## 4. Study Area and Methodology

In this section, we describe the geographic description of the seven cities used for experiments in this paper. Then we discuss the architectural differences among FCN-8, U-net, and DeepLabv3++.

### 4.1. Geographic Details of Seven Cities

In this study, we selected a city from each developing region of the “Global South” and classified the urban environments of these cities. The seven cities selected by regions are Nairobi (Sub-Saharan Africa), Mumbai (South Asia), Dhaka (South Asia), Jakarta (Southeast Asia), Guangzhou (East Asia), Cairo (North Africa), and Lima (Latin America). The cities were randomly chosen from different continents. The only criteria of the selection were that the cities had to be in the emerging economies of the global south. We used Google’s 50 km×50 km satellite image to draw a four-color map of the urban environment classification of the seven cities. The uninhabited area is marked with black. Each city has similarities and distinctive features. We included a short demographic description of the cities relevant to this research in Appendix A for the readers’ interest.

### 4.2. Deep Learning Algorithms for Automatic Building Categorization

This section briefly discusses different deep learning-based semantic segmentation algorithms and their applicability using satellite images. To automate the categorization method of [2], we examined three state-of-the-art algorithms that are widely used in satellite image data: FCN-8 [21], U-Net [22], and DeepLabv3+ [12]. We chose these particular models from three different families. FCN-8 was chosen from the VGG-16 family, where the encoder part was unchanged, but the decoder’s fully connected layers were replaced. Instead of upsampling the dense information directly, the output of the encoder (dense information) was merged with the other two encoder layer outputs and then upsampled. On the other hand, the decoder of U-Net takes the outputs of all encoder layer outputs and upsamples sequentially. DeepLabv3+ introduces atrous convolutions.

#### 4.2.1. Fully Convolutional Network (FCN-8)

**Figure 4:**
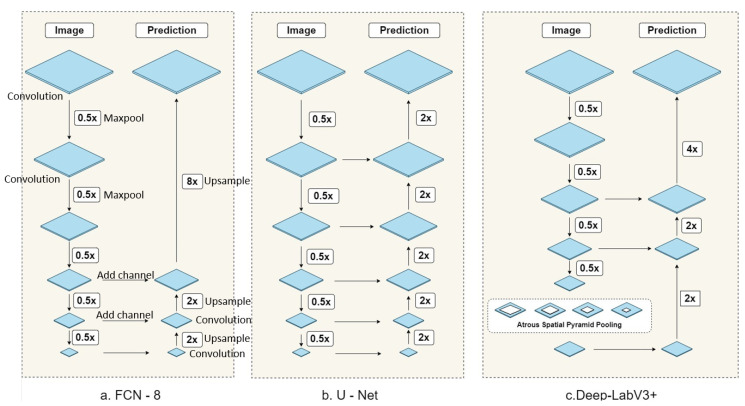
Architectures of (**a**) FCN-8, (**b**) U-Net, and (**c**) DeepLabv3+. The left downward flow of each model indicates the encoder part where the input is the three-channel color image. The right upward flow refers to the decoder part that outputs the final segmentation. Blue parallelograms indicate convolution operations; horizontal arrows from left to right show the channel additions from the encoder layers to the decoder layers; 0.5× boxes with the down-arrows refer to the max pool operations; 2×/4×/8× boxes with the up-arrows indicate the upsampling operation by 2, 4, and 8 factors, respectively. This figure is modified from [61].

FCN-based methods [21] have been used for classical segmentation tasks in the past couple of years. Several works use FCN–8 variants, which have higher precision in segmenting multisensory remote sensing images by using the features extracted from multiple spectral bands instead of FCN-16 and FCN-32; remote sensing images contain coarse, large objects, and small essential details. The algorithm uses the VGG-16 (Simonyan and Zisser man et al. [48]) as the backbone and replaces the fully connected layers with fully convolutional layers. This architecture shown in Figure 4a is the first deep segmentation model and has been widely used for object segmentation. Due to the use of many upsampling filters, this model has many parameters to be learned. However, this produces segmentation with coarse boundary due to loss of information in the downsampling process that induces unacceptable errors in satellite image segmentation. These images are patches of a larger image and, thus, will need to be stitched back together after testing where these errors accumulate. Additionally, overlapping outputs of the transpose convolution operation can cause undesirable checkerboard-like patterns in the segmentation map.

#### 4.2.2. U-Net

The U-Net segmentation model is developed by [22], based on the idea of FCN. Its architecture is similar to VGG-16, but with encoder–decoder architecture. It has divided into three parts. As shown in Figure 4b, the model skips connections between upsampling and downsampling paths, in order to provide local and global information during upsampling. Finally, at output 1×1, the convolutional layer provides the segmented output, where the number of feature maps is similar to the number of desired segments. This architecture has an improved boundary delineation and fewer parameters to work with. However, as the decoder cannot use dense information from different layers of encoders, the urban area segmentation from satellite images suffers from significant errors for some classes as opposed to doing well in others. This issue is further discussed in Section 6.

#### 4.2.3. DeepLabv3+

FCN-8 and U-Net are widely used for object segmentation, where objects are captured from a close distance. Examples of such scenarios are object segmentations for automatic driving cars, scene segmentation, etc. However, segmenting urban areas requires multiscale feature extraction that captures the urban building shapes, sizes, and textures, from different points of view. Successively, the extracted features and information help better urban segmentation. DeepLabv3+ [12] uses ImageNet’s pre-trained ResNet-101 with atrous convolutions as its main feature extractor. In the modified ResNet model, the last ResNet block uses atrous convolutions with different dilation rates. Moreover, it uses the Atrous Spatial Pyramid Pooling (ASPP) and bilinear upsampling for the decoder module on top of the modified ResNet block. The overall architecture has two main modules: encoder and decoder. The encoder is based on an output stride (ratio of the original image size to the size of the final encoded features) of 16. In the encoder, the ResNet-101 convolution layers are used. Moreover, the ASPP is exploited in the encoder to fuse features in different scales. In the decoder, instead of using bilinear upsampling with a factor of 16, the encoded features are first upsampled with a factor of 4 and concatenated with corresponding low-level features from the encoder module having the same spatial dimensions. Before concatenating, 1 × 1 convolutions in the decoder are applied on the low-level features to reduce the number of channels. After concatenation, a few 3 × 3 convolutions are applied, and the features are upsampled by a factor of 4. This gives the output of the same size as that of the input image.

Urban buildings with different shapes and textures define the urban environment. In order to categorize them effectively, the deep learning models should see the satellite images from different distances that allow the model to extract useful features. For example, analyzing the buildings from categories 2B and 3A from a fixed distance might not allow us to see the differences. However, examining them with different scales would help extract essential features useful to categorize them correctly. However, FCN-8 and U-Net do not have the capability to extract features with different scales. DeepLabv3+ model shown in Figure 4c has some unique modules that would help efficient and correct segmentation of satellite images. Atrous convolution with different dilation rates enable the model to extract image features in different scales. Specifically, the ASPP helps the model see satellite images from a different point of view. Analyzing the satellite images from a different view is crucial for good segmentation. ASPP helps the DeepLabV3+ model extract features from different levels, ranging from low-level pixel data to high-level contextual information. Figure 4 shows the architecture differences between these three algorithms and the efficacy of DeepLabv3+. Additionally, as the satellite images are captured from the top of the Earth, DeepLabV3 would show superior performance in segmenting the top view of the urban buildings and environment [61].

## 5. Automatic Building-Categorization

### 5.1. Data Collection, Annotation and Preprocessing

We use the freely available Bing satellite imagery for our building categorization task. Satellite images of the seven metropolitan areas were downloaded at a zoom level of 17, which has a spatial resolution of 2.26 meter/pixel. Even though the city administrative boundaries are not of a perfect rectangular shape, we produce an estimated rectangular shapefile of the seven cities by using its respective administrative shapefile for the sake of simplified grid prepossessing. Images are downloaded with geolocation metadata so that further analysis by georeferencing them can be done later. ArcMap GIS software was used to superimpose the shapefile of rectangular boundary and crop the region of interest (ROI) from the downloaded images. Downloaded data is annotated with ArcMap at various map scales to correctly identify each urban category within the context of a given city. Each city has a slightly different set of definitive features for urban classification. For example, the fully informal region from Dhaka is not quite similar to that of Cairo; since Dhaka has some vegetation between slums and Cairo is mostly a desert area. These customized definitions were considered during the annotation process. GeoTIFF images were converted to PNG in the data preprocessing stage, and patches of 513×513 were generated from the full-sized PNG image. This procedure is explained in detail in the following subsection. As for the annotations, the vector polygon layers are converted to raster images where the class labels are one-hot encoded to the pixel values as 0, 1, 2, 3, 4; where 0 is unclassified, and 1 to 4 represent classes ranging from fully informal to fully formal, respectively.

### 5.2. Producing Training Data with 70% Overlap

The convolution layers of the DeepLabv3+ are pre-trained with ImageNet. However, the Imagenet is the dataset of objects. In order to use the model for satellite image segmentation, we need to fine-tune the model weights by applying a sufficient training set. For each city, we trained a separate model with 70% of the corresponding city.

**Figure 5:**
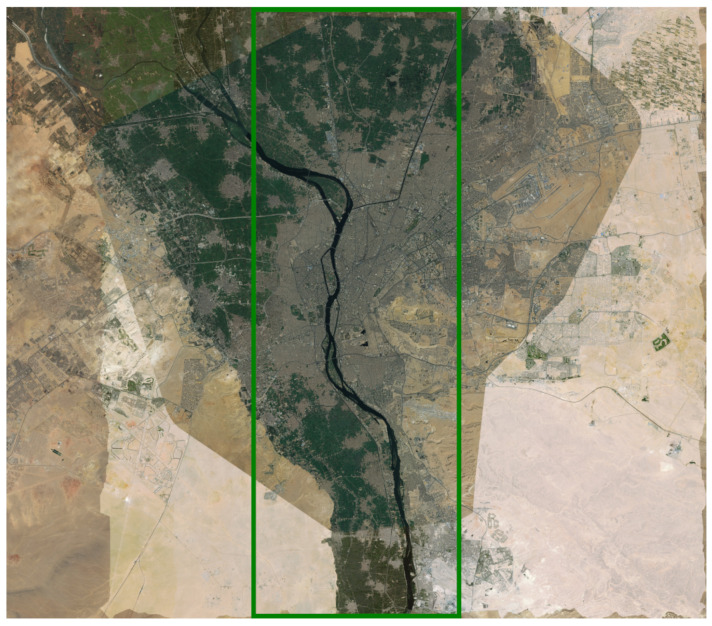
Input Satellite image for Cairo—Source: BING.

**Figure 6:**
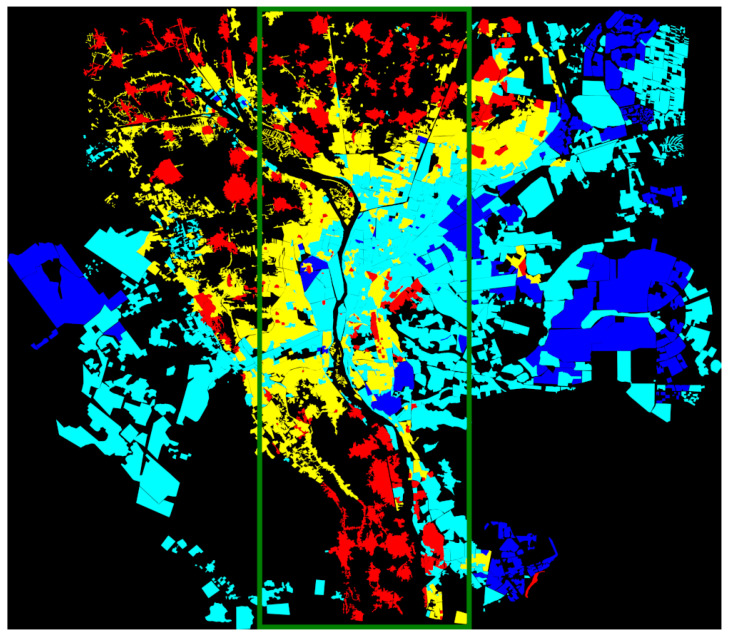
Ground truth for building categorization in Cairo. Red: highly informal area (arrival city); green: moderate informal area; cyan: moderate formal area; blue: formal area; black: uninhabited area.

**Figure 7:**
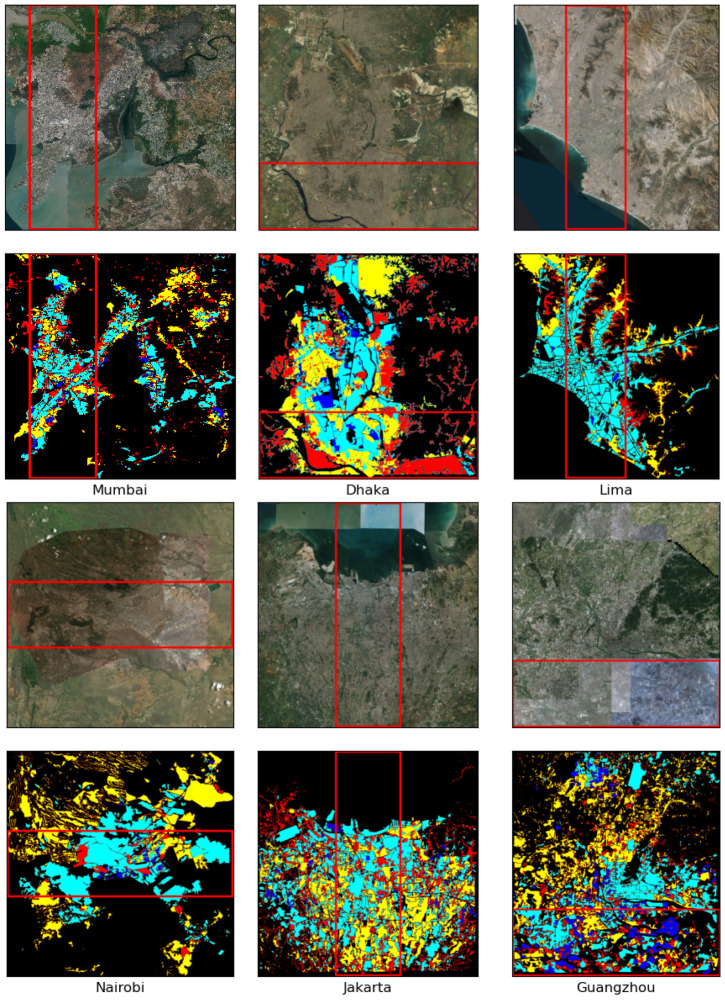
Input images and ground truths for other six cities. Red: highly informal area (arrival city); yellow: moderately informal area; cyan: moderately formal area; blue: highly formal area; black: uninhabited area.

We split the input image and the corresponding ground truth into two parts—one part for the training and the other for the testing. The training part consists of the 70% of each city image. For example, the 70% portion of the Cairo outside of the middle red rectangle shown in Figure 5 has been used for training, and the middle 30% area is used for testing. Figure 6 shows the ground truth image for Cairo. Figure 7 shows the satellite images and ground truths for the other six cities. The red rectangles define the test area, whereas the area outside the rectangles is used to train each deep model for the corresponding city.

Before feeding the image into the network, some required pre-processing of the data was needed. In the first step, both the input image and its target image needed to be in a grid of 513×513, as it was the required width and height to feed into a segmentation model. However, this grid was built using a 70% overlap of the previous image. This 70% overlap grid was made both vertically and horizontally. Overlapping increases the training data drastically. At the next step, the ground truth was converted into an index image.

Index images are single-channel images where the class value is one-hot encoded to the corresponding pixels. Again, this is done because it is required to feed into a model. Afterward, mapping of the input image was done using the ground truth image data. We projected the uninhabited part on the input image to prevent misclassification. After all the pre-processing was done, the images were fed into the particular model.

### 5.3. Training Procedure

#### 5.3.1. Training

Training was done separately for each of the cities with three different models. When the training data were ready, we fed the data into each of the three models. Each model was initialized with pre-trained ImageNet weights to reduce training time by quite a lot. A total of 7×3=21 (7 cities with three deep models) experiments were done for this paper.

#### 5.3.2. Hyperparameters

Then, we calculated weights over the total number of classes for balancing the class weights. We used stochastic gradient descent (SGD) as our optimizer. The dataset had 5 classes, including the unlabeled/unrecognized area. A learning rate of 0.007 was used over 26 epochs.

### 5.4. Testing Procedure

For the test data, we used 30% of the total city area. The red rectangles of the Figure 5 and Figure 7 refer to the test parts for each corresponding city. The same pre-processing method was done, but during the grid and stitch, no image overlapping was done since it would only be used for testing.

### 5.5. Performance Evaluation

After the testing was done, a particular model generated output images. Those output images were later converted into RGB images for both performance calculation and visual representation. All images were then stitched to their original dimensions.

We compared the output images with the original ground truth images to calculate accuracy, F1score. Accuracy is calculated by summing the number of correctly classified pixel values and dividing by the total number of pixel values. Accuracy helps us to understand what percentage of the segment is correctly ascertained. The F1 score is the harmonic mean of recall and precision where recall is measured to ascertain what percentage of positive pixels are predicted. Precision is the measure that helps to ascertain what percentage of relevant pixels are predicted. The F1 score allows us to quantify the performance of the segmentation for imbalanced samples.

## 6. Experimental Results and Performance Comparison

In this section, we discuss the results of our experiments. First, we show the best performance from DeepLabv3+ over the test part of Cairo city. We explain the performance variations with accuracy and the F1-score. Then, we discuss the detailed performance comparison over seven cities with DeepLabv3+, U-Net, and FCN-8. We conclude this section by providing the performance summary.

**Figure 8:**
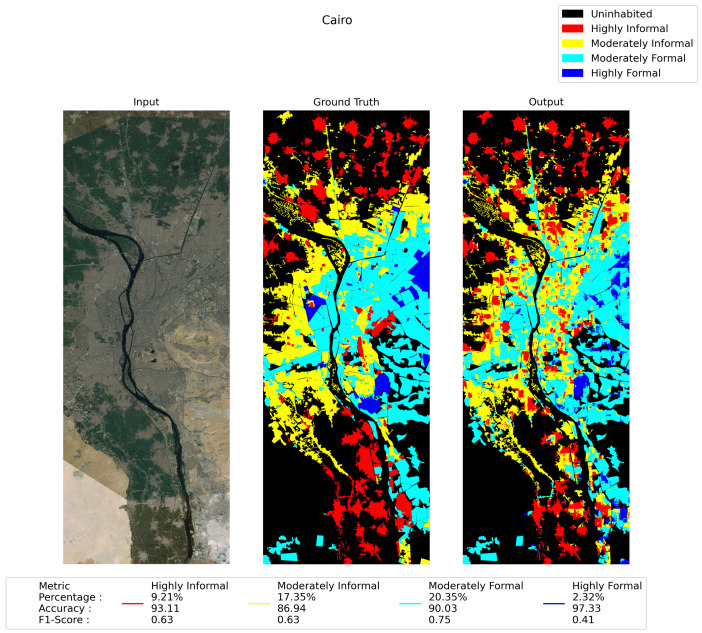
Test image, ground truth, and the test output—testing with 30% vertically middle part of Cairo.

The Figure 8 depicts the performance of the segmentation for Cairo city over the test area using DeepLabv3+. The left sub-image is the test image that was used as the input to the trained model. The right-most sub-image is the output from the segmentation model. The middle one is the ground truth image, where different colors represent different building categories.

We notice that the model can segment the highly informal area with 93.11% accuracy. The moderately informal area is a little bit difficult and error-prone compared to other classes. Various building material usage, irregular shapes and sizes, and no fixed building codes are the main factors that cause the model to categorize the informal area with a comparatively lower accuracy and F1-score. For example, this area consists of buildings with roofs with corrugated tin, concrete, bamboo, or even hardboards. Due to having very similar surface morphology, the moderately informal and highly informal areas are misclassified as each other. However, the moderately informal area has been segmented with reasonably high accuracy: 86.94%. On the other hand, moderately formal areas can be segmented effectively with 90.03% accuracy, with an F1-score of 0.75. Finally, the DeepLabv3+ could segment the highly formal area with an accuracy of 97.33% and an F1 score of 0.41. An important characteristic of the highly formal area that contributed to a comparatively lower F1 score across the models is the highly formal area being intertwined with other classes. Mosques, parks, shopping centers, and highly formal housing projects often sprung up in the middle of a largely informal or moderately informal urban setting. A very insignificant percentage area—below 10% for Cairo city, Figure 8 in the train-set, made this issue worse. However, the deep learning model can categorize urban areas with an average of 91.85%.

For the other six cities, the red rectangles in Figure 7 represent the test areas for corresponding cities. We selected the test rectangle so that all class representative pixels are present in the test area. Table 2 lists the detailed performance comparison for the segmentation on seven cities with all three models (DeepLabv3+, U-Net, and FCN-8). The table shows that DeepLabv3+ performed better than U-Net, and significantly better than FCN-8, in terms of accuracy and F1 score in all seven cities for segmenting the four socioeconomic areas. The only exceptions are: highly informal and moderately informal areas in Guangzhou—U-Net performs a little bit better than DeepLabv3+ in terms of accuracy and F1 score. In Nairobi, highly informal and highly formal areas are segmented by U-Net with marginally better accuracy compared to DeepLabv3+. However, the F1 scores for U-Net for these areas are significantly lower than those of DeepLabv3+.

Careful observation reveals that each city has a very small portion of the highly formal area and has been segmented with the highest accuracy, despite having the lower F1-score. These phenomena are contributed by the couplings between nearby class areas and the imbalance present throughout the test-set for each city where the highly formal area is below 3%, even though the percentage area is a bit higher in the train set (up to 10%). For Mumbai, Lima, Jakarta, Guangzhou, and Nairobi, we can observe this issue clearly if we look at the area percentage in Table 2, as well as Figure 7, where regions marked in blue represent the highly formal areas. DeepLabv3+ did better than others, as we can see from its F1 scores, where U-Net and FCN-8 failed miserably (F1 scores shown in italic) in segmenting highly formal areas. On a moderately skewed satellite imagery dataset where classes are very similar at different map scales, models such as FCN-8 yield catastrophic results for less represented classes. On the other hand, Cairo and Guangzhou have comparatively higher percentage areas in the test-set as highly formal. These two cities are more dynamic and have seen rapid development in recent years. Cairo specifically has distinctive shapes in its highly formal structures, similar to that of the neighboring developed Arab nations. Mostly informal to moderately formal urban settings were developed on the bank of the river Nile throughout the ages of its very long history. Highly formal areas were developed more recently on the outskirt of the city in the desert area. These formal regions are more recognizable through human eyes than the other parts of the region.

Additionally, we can see that in Guangzhou, the accuracy of moderately informal and moderately formal regions is the lowest by DeepLabv3+, U-Net, and FCN-8. Guangzhou has many rivers and waterways supporting the dynamic and sprawling urban nature of the port city situated on the northwest of Hong Kong. Here, frequently, two sides of a river have two polar opposite urban settings. It can be seen from the map that many slums in Guangzhou are mixed in dense and complex terrain where slums are spread over the entire map and evenly distributed in industries and areas adjacent to industries. The diverse and sporadic regions are complex and are found difficult for the segmentation model to learn. Looking deeper into the issue, we observe that the most used roofing material is tin, and in highly informal areas, they are very rusty, more often than not. These may trick an insincere observer into believing that there is some gap between the slums; thus, adding further complexity to our categorization task.

In summary, the average accuracy and average F1 scores across all cities and all classes for DeepLabv3+ are 91.78% and 0.55. On the other hand, the average accuracy and average F1 score for U-Net and FCN-8 are 90.35%, 0.46, 88.46%, 0.39, respectively. Hence, it is easy to notice that DeepLabv3+ performs better for socioeconomic segmentation tasks.

Table 3 compiles the summary of the performance of the models on the corresponding socioeconomic area across all cities. We calculated the average F1 score and standard deviations of the F1 scores across all cities for each model and placed them here. It can be easily observed that DeepLabv3+ has the best F1 scores for four areas. Furthermore, FCN-8 has the least F1 scores. The results suggest that moderately formal areas seem to be segmented more accurately than the other classes with all three models. The second-best performance was achieved for highly informal areas. Similar performance were observed for the moderately informal area. As discussed in the data imbalance issue for the highly formal area in earlier paragraphs, all three models achieve the least average F1 scores to segment this area. Large standard deviations of F1 scores indicate that the different kinds of urban environments and building materials cause the deviation of model performances across different cities. For example, different building materials, cultures, traditions, and geographic locations make the highly informal urban area versatile while analyzed from a satellite image. The observations are supported by similar standard deviations (0.20, 0.23, and 0.21) for the highly informal areas across the three models: DeepLabv3+, U-Net, and FCN-8, respectively. This means the models can capture the diversities across cities equally. Similar kinds of patterns are observed for other areas too.

## 7. Policy Implication and Conclusions

This research has demonstrated how a novel categorization method for urban classification can be automated using a deep learning model. The framework proposed was compared among seven different cities of the global south. Satellite data have become a popular means of policy analysis due to the advent of computational power and image processing techniques using deep learning. Our proposed method is proven to be accurate, readily usable, and scalable, especially for complex urban areas of the developing world. Compared with other globally available statistics, the framework is physically comparable and can be conducted in greater frequency, enabling the capacity to monitor the urban environment at run time.

This study expands our understanding of the morphological characteristics of various urban environments around the world.

We used a novel categorization method proposed in [2], which is readily applicable for deep learning mechanisms for urban data classification. Based on the definitions of formal and informal areas in the developing world’s urban setting provided by the authors, we categorized urban satellite images of seven different cities of the developing world. We divided the urban environment into 16 categories through the two axes of architectural form and the urbanization process, and mapped an extensive range (50 × 50 km) of urban environments based on satellite images and visual recognition methods. The carefully picked (70%) image data for each city was used for training and the rest for test purposes.

DeepLabv3+ segments the four socioeconomic areas with accuracies of 90.0% for Dhaka, 91.5% for Nairobi, 94.75% for Jakarta, 82.0% for Guangzhou city, 94.25% for Mumbai, 91.75% for Cairo, and 96.75% for Lima. However, the performance varies across different cities due to architectural and texture differences among them. In Cairo, both the formal and informal areas include brick buildings. On the other hand, in Nairobi, most of the building materials in the informal areas are made of iron sheets and hardboards with tin roofs on top, whereas the formal area has concrete roofs.

Mode, quantity, and form as the basis of urban geography and urban poverty research: this categorization method allows us to match spatial knowledge with urban location information of possible locations, combine topography, area, population density, income, and education data, and adopt a more systematic and consistent method to locate the urban population globally. It can even complete a morphological catalog of the global urban environment. 

## Figures and Tables

**Table 1:** Selection of colors for urban environment typology for Cairo.

Urban Environment Topology	Category	Color
Red (1/A, 2/A, 2/B)	Highly informal	
Yellow (1/B, 1/C, 1/D, 2/C, 2/D, 3/A)	Moderately informal	
Cyan (3/B, 3/C, 3/D, 4/A, 4/B, 4/C)	Moderately formal	
Blue (4/D)	Highly formal	

**Table 2:** Detailed performance of segmentation over six cities.

			DeepLabv3+		U-Net		FCN-8	
**City**	**Socioeconomic** **Semantic Region**	**Area** **(%)**	**Accuracy** **(%)**	**F1-Score**	**Accuracy** **(%)**	**F1-Score**	**Accuracy** **(%)**	**F1-Score**
Dhaka	Highly Informal	20.86	86.47	0.66	85.72	0.65	80.52	0.59
	Moderately Informal	17.95	83.24	0.56	81.74	0.55	79.11	0.34
	Moderately Formal	12.28	94.33	0.77	93.95	0.78	89.01	0.59
	Highly Formal	0.95	99.10	0.18	98.98	0.05	98.20	0.11
Mumbai	Highly Informal	7.13	98.10	0.86	97.27	0.77	95.12	0.67
	Moderately Informal	8.29	91.16	0.45	88.94	0.36	89.53	0.26
	Moderately Formal	16.56	90.25	0.71	89.34	0.69	88.09	0.66
	Highly Formal	2.08	98.0	0.10	97.75	*0.02*	97.74	*0.01*
Lima	Highly Informal	4.81	98.05	0.81	97.32	0.79	96.43	0.56
	Moderately Informal	8.51	95.14	0.73	89.42	0.54	89.54	0.49
	Moderately Formal	33.20	95.03	0.93	90.48	0.86	89.32	0.85
	Highly Formal	0.72	**99.01**	0.17	99.18	*0.01*	99.20	*0.00*
Jakarta	Highly Informal	9.78	95.20	0.73	92.31	0.50	86.52	0.38
	Moderately Informal	19.25	91.02	0.79	84.81	0.63	78.69	0.43
	Moderately Formal	28.13	94.01	0.89	89.04	0.81	82.59	0.69
	Highly Formal	0.64	99.05	0.22	99.21	*0.03*	99.04	*0.01*
Cairo	Highly Informal	9.21	93.11	0.63	90.89	0.42	89.08	0.40
	Moderately Informal	17.35	86.94	0.63	84.07	0.58	83.58	0.54
	Moderately Formal	20.35	90.03	0.75	88.79	0.73	87.93	0.70
	Highly Formal	2.32	97.33	0.41	96.74	0.24	96.32	0.33
Guangzhou	Highly Informal	11.88	90.31	0.32	**90.70**	**0.40**	88.80	0.13
	Moderately Informal	13.19	71.11	0.36	**73.03**	**0.36**	76.30	0.28
	Moderately Formal	21.08	77.05	0.43	74.20	0.42	70.29	0.47
	Highly Formal	10.82	90.18	0.25	89.40	*0.06*	89.05	*0.05*
Nairobi	Highly Informal	3.6	94.04	0.41	**96.34**	0.16	96.11	0.16
	Moderately Informal	13.28	87.12	0.58	85.62	0.59	81.14	0.46
	Moderately Formal	32.55	87.08	0.77	86.41	0.77	81.50	0.69
	Highly Formal	1.91	98.00	0.19	**98.14**	*0.00*	98.12	0.00

**Table 3:** Performance summary for DeepLabv3+, U-Net, and FCN-8.

	DeepLabv3+ Average F1	DeepLabv3+ Stdev (F1)	U-Net Average F1	U-Net Stdev (F1)	FCN-8 Average F1	FCN-8 Stdev (F1)
Highly Informal	0.63	0.20	0.53	0.23	0.41	0.21
Moderately Informal	0.59	0.15	0.52	0.11	0.40	0.11
Moderately Formal	0.75	0.16	0.72	0.14	0.66	0.12
Highly Formal	0.30	0.23	0.16	0.28	0.14	0.23

## Data Availability

Not applicable.

## Appendix A

### Appendix A.1. Dhaka

The urban population of Bangladesh has increased from 7.6% in 1970 to 37.4% in 2019. The increasing urban–rural migration in Dhaka makes urban governance and proper resource allocation more difficult. Our 50 × 50 km map includes Dhaka city and its surrounding areas, such as Tongi, Keraniganj, Zazira, Matuail, and Rupganj. The informal settlements in Dhaka city are mainly distributed around the basin of the Buriganga River and the Shitalakshya River. The main building materials are sheet iron, wood, brick, paper, etc. Formal areas are distributed near the airport.

### Appendix A.2. Cairo

The average population density of the Greater Cairo metropolitan area is 6667 per square kilometer. It covers an area of about 17,267.6 square kilometers. Greater Cairo is divided into Cairo, New Cairo, Giza, Shubrā al-Khaymah, the 6 October (city), Helwan, and El-Obour As a result, our 50 × 50 km map includes the above areas and some suburbs. As desert areas surround Cairo, high-density residential areas are distributed in the Nile River Basin. Informal areas are mainly distributed on both sides of the Nile River Basin, as well as the farmland around the north, west, and south. The formal area is located in the core area of Cairo’s new town and Giza. The buildings in Cairo’s informal areas are mainly made of bricks.

### Appendix A.3. Nairobi

The 50 × 50 km map, centered on Nairobi, Kenya, contains surrounding cities, such as RuiRu, Kiambu, Ruaka, Ndenderu, Nyathuna, Gitaru, Kikuyu, Muguga, Ngong, Kiserian, Ongata Rongai, Tuala, Mlolongo, Pridelands, Ngelani city, and Githunguri. The population of this area is about 4,897,073. Due to rapid population growth and urban poverty, most urban settlements are informal. Unofficial areas in Nairobi are mainly distributed along the Nairobi River, and Gong River in the city center, near factories along the railway; several large unofficial areas are scattered in some green spaces and undeveloped areas. It can be seen that in the vicinity of the large development zone in the southwest, informal areas have solidified. There are few informal areas on the outskirts of Nairobi. Most of the building materials in these informal areas are made of iron sheets and formwork. At the same time, there are official areas in the center of Nairobi.

### Appendix A.4. Lima

Lima is the capital and the largest city of Peru. The main urban area is located in the basin of the Cheyne, Rímac, and Luen rivers, which extend from the central coastal plain to the Pacific Ocean. With the complex geographical features of oceans, mountains, and deserts, the current capital Lima includes the central area with formal structure and the "cone", the peripheral area with the informal area. Our 50 × 50 km maps cover the Lima District, San Martin de Porres, Los Olivos, Ventanilla, Comas, Puente Piedra, Carabayllo, San Juan de Lurigancho, Ate, La Molina, Santiago de Surco, Chorrillos, Villa El Salvador, and Villa Maria Del Triunfo. Moreover, 72% of households in Lima live in the expansion of informal areas, without adequate housing and proper sanitation. Most of these informal areas are located in green and undeveloped areas. These informal areas are close to the slope and extend northward and eastward from the city center. They radiate through the whole city and are distributed in three cones. Most of the houses in these informal areas are made of bricks and iron sheets.

### Appendix A.5. Jakarta

Jakarta is on the coast of Java. It has a population of more than 30 million and a total area of 4384 square kilometers. Rapid economic growth leads to large-scale urbanization. The population growth of the Jakarta metropolis is the main reason for the rise of informal settlements. Our 50 × 50 km map mainly contains five major cities of Jakarta Metropolis: central Jakarta (Jakarta Pusat), Western Jakarta (Jakarta Bharat), southern Jakarta (Jakarta Selatan), Eastern Jakarta (Jakarta Timur), and Northern Jakarta (Jakarta Utara). In Jakarta, people often see informal areas spread around the old city; informal areas are concentrated in industrial areas and industrial areas near the northern coast. These informal settlements are located in the northern part of Jakarta, near the Ciliwung Estuary, in the areas of Pasar Baru, Jakarta, and Mangga Dua. The most common building materials are wood, bamboo, cardboard, and plastic.

### Appendix A.6. Mumbai

Mumbai is the capital of Maharashtra. It covers an area of 6355 square kilometers and has a population of more than 26 million. Our 50 × 50 km map includes Fort, Mumbai, Bhandup West, Andheri West, Thane, Gorai 2, and Mira-Bhayandar. The city center of Mumbai is located near the top of the peninsula. Many dense informal settlements in Mumbai are mainly distributed along the traffic lines and in vulnerable areas—for example, near rivers, natural parks, wetlands, green space, and undeveloped land. The building materials of these informal settlements are generally iron plates and wood. In addition, the official areas of Mumbai are distributed in the urban centers of Colaba, Fort, Dadar, Andheri East, and others.

### Appendix A.7. Guangzhou

Guangzhou covers an area of about 7434 km${}^{2}$. In the past 20 years, large-scale population migration has taken place in the urban area of Guangzhou. The resident population increased from 5.9 million (1990) to 15.3 million (2019). The increase in population leads to the expansion of urban areas. Our 50 km × 50 km map includes Guangzhou and Foshan. It can be seen from the map that many informal areas in Guangzhou are mixed in dense and complex terrain. In Guangzhou, informal areas are all over the map, evenly distributed in the industrial and adjacent areas. Moreover, there are many informal areas in the south of the city center, large development zones, and the north of the city center. Slums can also be found in the northern suburbs of cities and in rural areas. The building materials of these informal settlements are mainly brick and concrete.
